# Investigating the effect of positional variation on mid-lactation mammary gland transcriptomics in mice fed either a low-fat or high-fat diet

**DOI:** 10.1371/journal.pone.0255770

**Published:** 2021-08-26

**Authors:** Adrienne A. Cheng, Wenli Li, Laura L. Hernandez

**Affiliations:** 1 Department of Nutritional Sciences, UW-Madison, Madison, Wisconsin, United States of America; 2 Department of Animal and Dairy Sciences, UW-Madison, Madison, Wisconsin, United States of America; 3 Cell Wall Biology and Utilization Research Unit, US Dairy Forage Research Center, Agricultural Research Service, US Department of Agriculture, Madison, Wisconsin, United States of America; University of Agricultural Sciences, INDIA

## Abstract

Little attention has been given to the effect of positional variation of gene expression in the mammary gland. However, more research is shedding light regarding the physiological differences that mammary gland location can have on the murine mammary gland. Here we examined the differentially expressed genes between mammary gland positions under either a low-fat diet (LFD) or a high-fat diet (HFD) in the mid-lactation mammary gland (lactation day 11; L11). Three-week old WT C57BL/6 mice were randomly assigned to either a low-fat diet (LFD) or high fat diet (HFD) (n = 3/group) and either the right thoracic mammary gland (TMG) or inguinal mammary gland (IMG) was collected from each dam for a total of 12 unique glands. Within each diet, differentially expressed genes (DEGs) were first filtered by adjusted *p-*value (cutoff ≤ 0.05) and fold-change (FC, cutoff ≥2). Genes were further filtered by mean normalized read count with a cutoff≥10. We observed that mammary gland position had a significant impact on mammary gland gene expression with either LFD or HFD diet, with 1264 DEGs in LFD dams and 777 DEGs in HFD dams. We found that genes related to snRNP binding and translation initiation were most significantly altered between the TMG and IMG. Although we were not able to discern a molecular mechanism, many small nuclear RNAs and small nucleolar RNAs were differentially expressed between the TMG and IMG responsible for cellular functions such as splicing and ribosome biogenesis, which provides and interesting avenue for future research. Our study supports the hypothesis that collection of the mammary gland from a particular location influences mammary gland gene expression, thereby highlighting the importance for researchers to be vigilant in documenting and reporting which mammary gland they are using for their studies.

## Introduction

Early studies focused on the mammary gland using a rabbit model led scientists to believe that mammary gland development was symmetrical, giving the impression that each gland was just a copy of the other [1, 2]. However, studies in the mouse have demonstrated that this is not the case [2–4]. It has recently become apparent that different signaling pathways occur in select mammary pairs [5]. There is also an increasing body of work demonstrating that mammary gland position affected tumor incidence and patient survival [2, 3]. This further highlighted the necessity to understand gene expression differences between different mammary glands in the rodent model.

In the mouse, the majority of mammary gland development occurs postnatally at approximately three weeks of age (puberty), and again during pregnancy and lactation [6]. Our lab is recently interested in the effects of diet during puberty, pregnancy, and lactation on lactating mammary gland function. Specifically, many of our studies have examined mammary gland changes during peak lactation and therefore is the focus of this experiment. Mice have ten teats (5 pairs) and include cervical, two thoracic, abdominal, and inguinal [7]. The main goal of our study is to utilize the right thoracic mammary glands (TMG) and the right inguinal mammary gland (IMG) to showcase the differences in mammary gland transcriptomics due to positional variation. In addition to understanding the effect of positional variation on global gene expression profile in the mammary gland, we sought to understand the commonality and differences in gene expression profile under different diets. Based on previously established data that diet can affect pubertal mammary gland development [8], in this study, we fed mice either a low-fat diet (LFD) or high-fat diet (HFD) beginning at three weeks of age through lactation. Therefore, the goal of this experiment was to determine global gene expression changes in the inguinal versus thoracic peak lactation mammary gland when fed either LFD or HFD.

## Results

### Differences in thoracic and inguinal mammary gland gene expression in peak lactation dams fed a LFD

**Fig 1:**
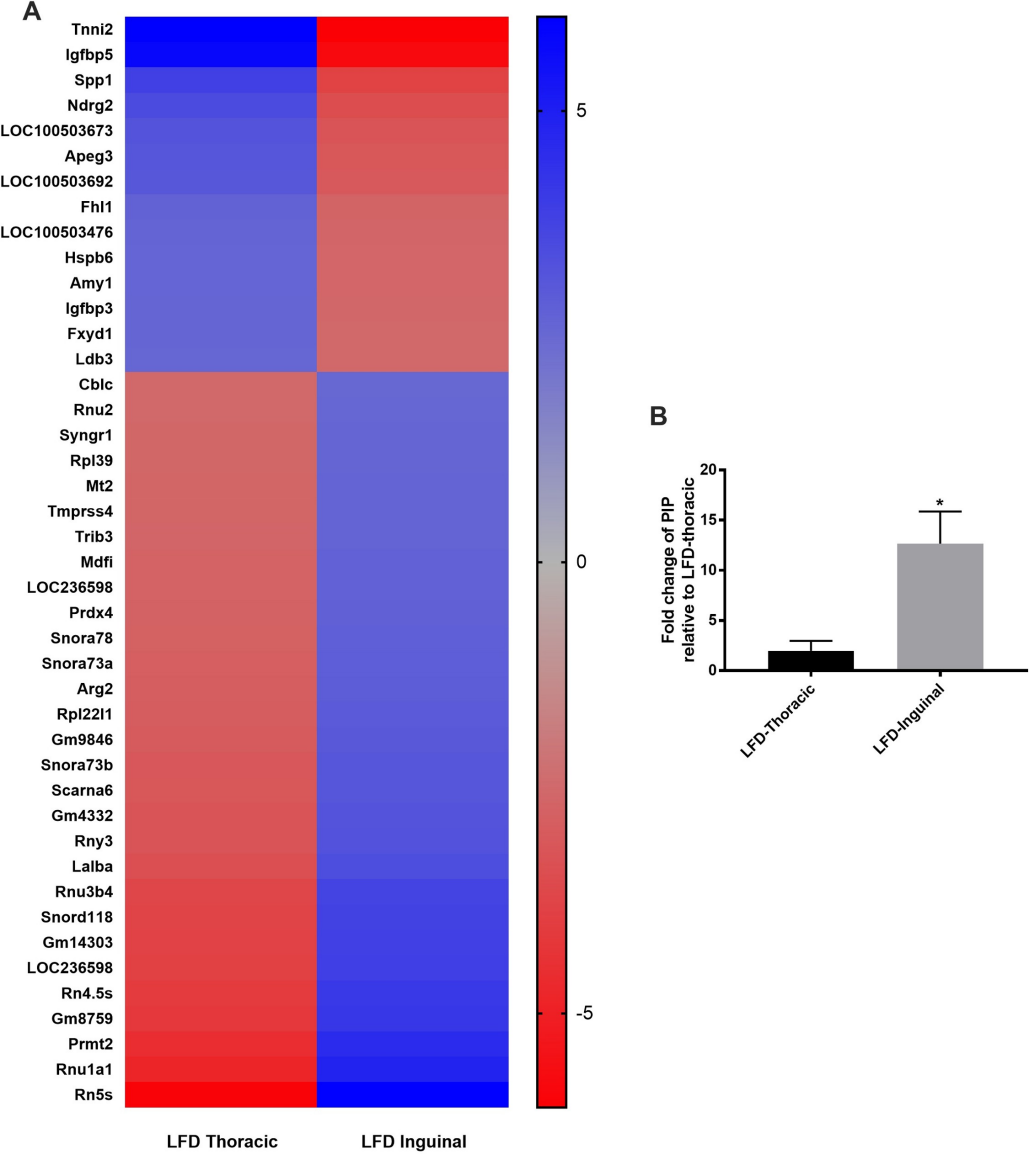
**A) Heatmap displaying the 43 top DEGs with a log2FC absolute value greater than or equal to 2.5 L11 dams fed a LFD. B) qRT-PCR verification of prolactin inducible protein (PIP); p = 0.034; one tailed t-test**.

**Fig 2:**
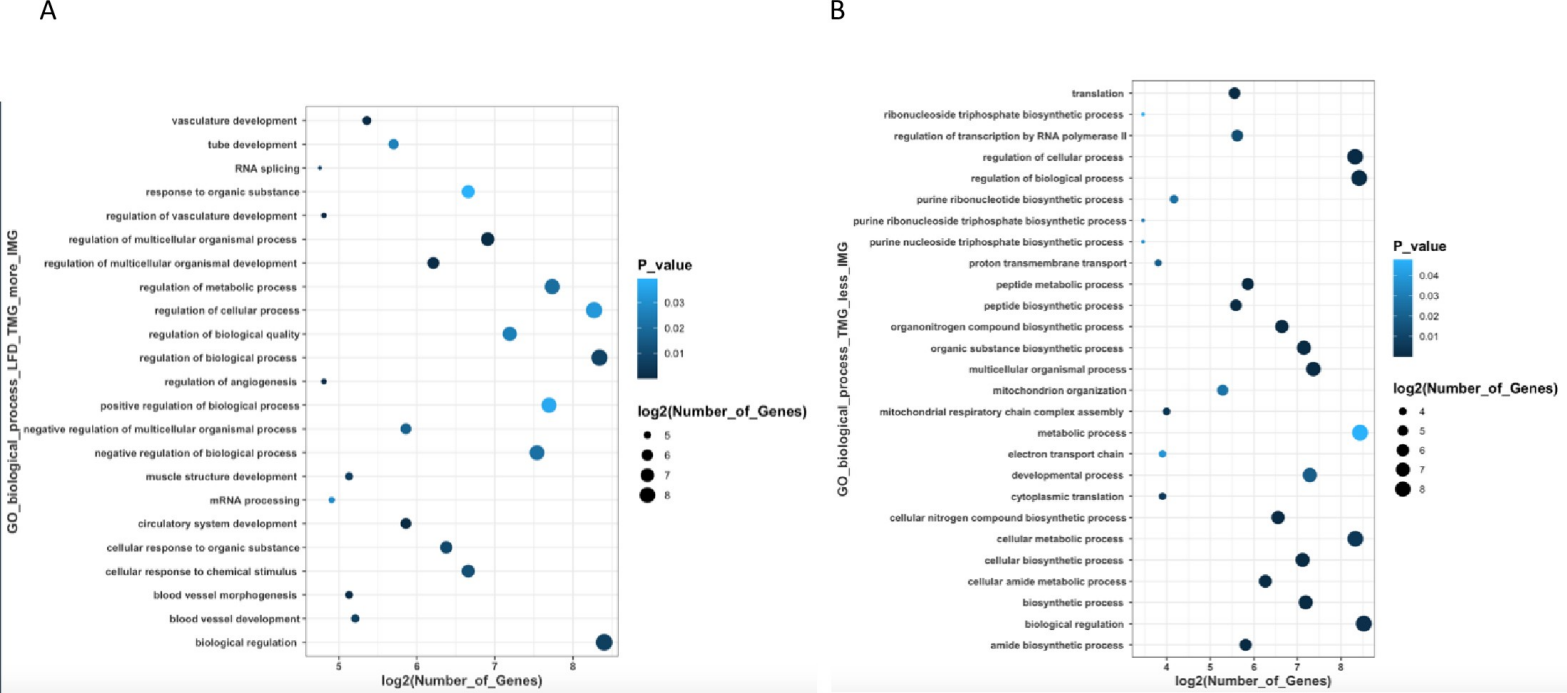
**Summary of GO Biological Processes that are most upregulated (A) in the thoracic mammary gland and downregulated (B) in the TMG in dams fed a LFD**.

A total of 1264 genes were differentially expressed between the TMG and IMG (**S1 Table**) in LFD fed dams. There were 503 genes upregulated in the TMG and 761 genes downregulated in the TMG when compared to the IMG. Notable biological processes that were upregulated in the TMG were those involved in circulatory system development (GO:0072359; p < 0.001), blood vessel morphogenesis (GO:0048514; p < 0.001), and RNA splicing (GO:0008380, p < 0.001) (**S2 Table**). Meanwhile, biological processes that were downregulated in the TMG when compared to the IMG included cellular amide metablic processes (GO:0043603; p <<< 0.0001), organonitrogen compound biosynthetic processes (GO:1901566, p << 0.0001), translation (GO:0006412; p <<< 0.0001), and amide biosynthetic processes (GO:0043604; p <<<0.0001). Other notable biological processes upregulated in the TMG included processes related to purine ribonuleoside and nucleoside biosynthetic processes (**S3 Table**). Molecular functions upregulated in the TMG included binding (GO: 0005488; p << 0.0001), mRNA binding (GO:0003729, p < 0.001), protein binding (GO:0005515, p < 0.01), and nucleic acid binding (GO:0003676; p < 0.01). (**S4 Table**). Molecular functions downregulated in the TMG included structural constituents of ribosome (GO:0003735; p <<< 0.0001), DNA binding transcription factor activity (GO:0003700; p < 0.01), and double-stranded DNA binding (GO:0003690; p < 0.01) (**S4 Table**) Reactome pathways that were downregulated in the TMG when compared to IMG included pathways involved in translation (R-MMU-72766; p <<< 0.0001), formation of pool of free 40S subunits (R-MMU-72689; p <<< 0.0001), and cap-dependent translation initiation (R-MMU-72737; p <<< 0.0001) (**S5 Table**). A summary of top DEGs and GO biological processes affected by mammary gland position in dams fed a LFD are depicted in **Figs 1 and 2**.

### Differences in thoracic and inguinal mammary gland gene expression in peak lactation dams fed a HFD

**Fig 3:**
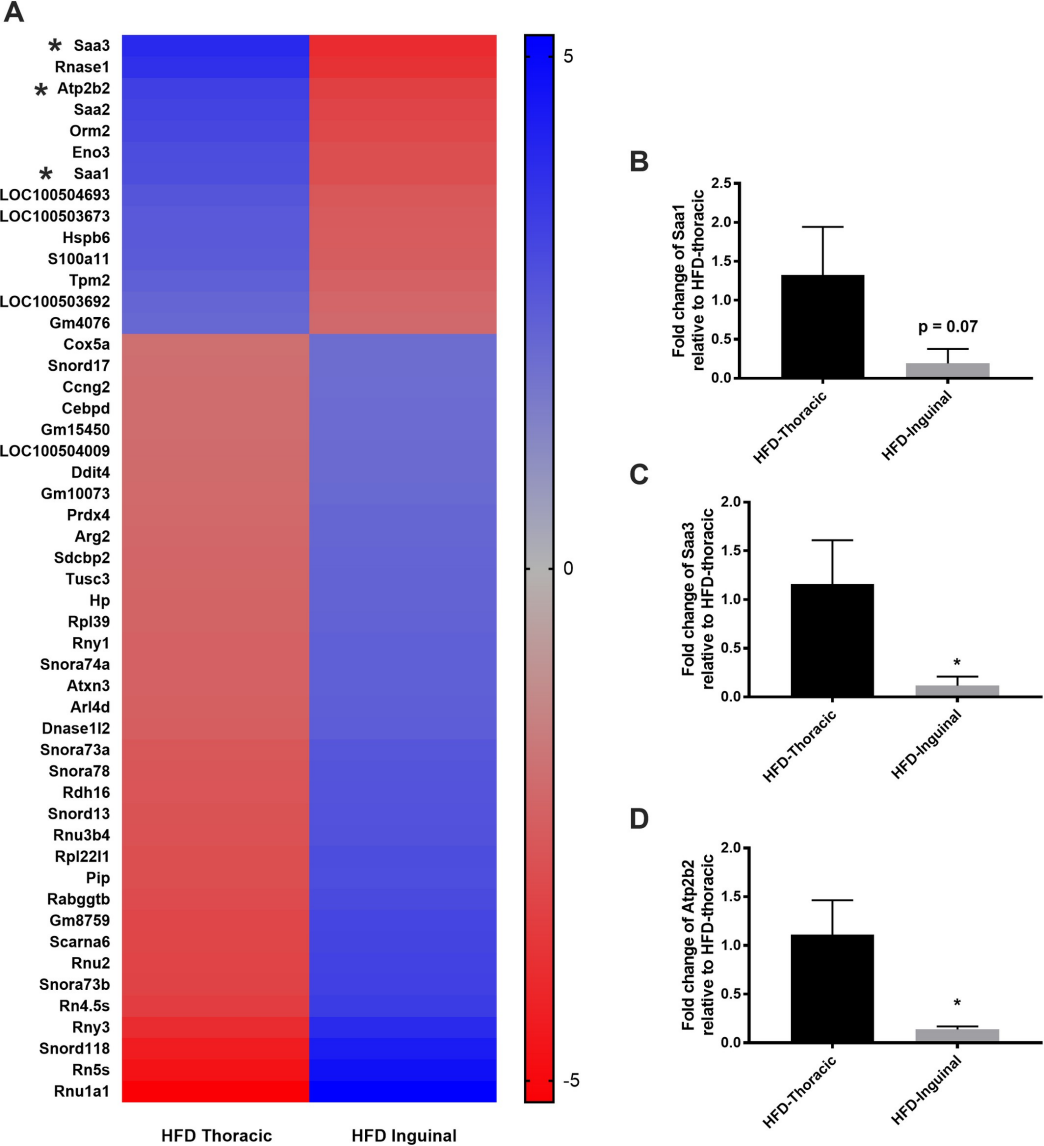
A) Heatmap displaying the 50 top DEGs with a log2FC absolute value greater than or equal to 2.0 in L11 dams fed a HFD. qRT-PCR verification of B) Saa1, C) Saa3, and D) Atp2b2; p < 0.05; two tailed t-test.

**Fig 4:**
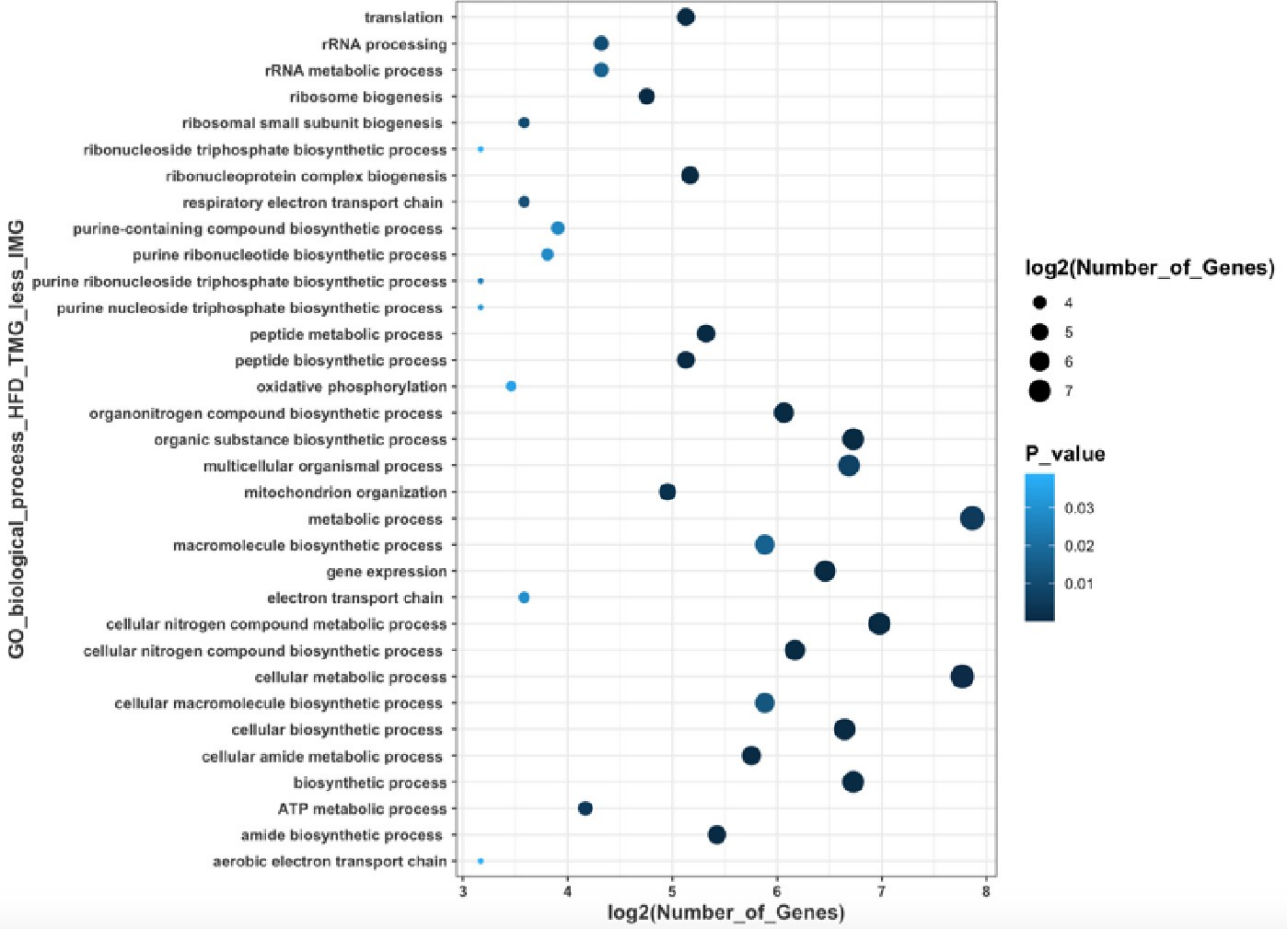
Summary of GO biological processes that are downregulated in the TMG of dams fed a HFD.

There were 777 DEGs in HFD dams between the TMG and IMG, 296 of which were upregulated in the TMG compared to the IMG, and 481 that were downregulated in the TMG compared to the IMG (**S6 Table**). No GO biological processes., molecular functions, or reactome pathways were significantly upregulated in the TMG compared to the IMG in dams fed a HFD. GO biological processes downregulated in the TMG compared to the IMG included those involved in amide biosynthetic process (GO:0043604; p <<< 0.001), translation (GO0006412; p <<< 0.0001), and peptide biosynthetic processes (GO:0043043; p << 0.0001) (**S7 Table**). Molecular functions significantly downregulated in the TMG compared to IMG included structural constituent of ribosome (GO:0003735; p <<< 0.0001), electron transfer activity (GO:0009055; p << 0.001), and RNA binding (GO:0003723; p < 0.05). Reactome pathways that were downregulated in HFD TMG dams compared to IMG included metabolism of RNA (R-MMU-8953854; 41 genes; *p* << 0.0001), translation (R-MMU-72766; p <<< 0.0001), and rRNA processing (R-MMU-6791226,R-MMU-8868773,R-MMU-72312; p <<< 0.0001) (**S8 Table**). A summary of the differences between TMG and IMG in peak lactation dams fed a HFD is depicted in **Figs 3 and 4**.

### Examination of the unique and shared pathways up and downregulated between the TMG and IMG in dams fed either a LFD or HFD

**Fig 5:**
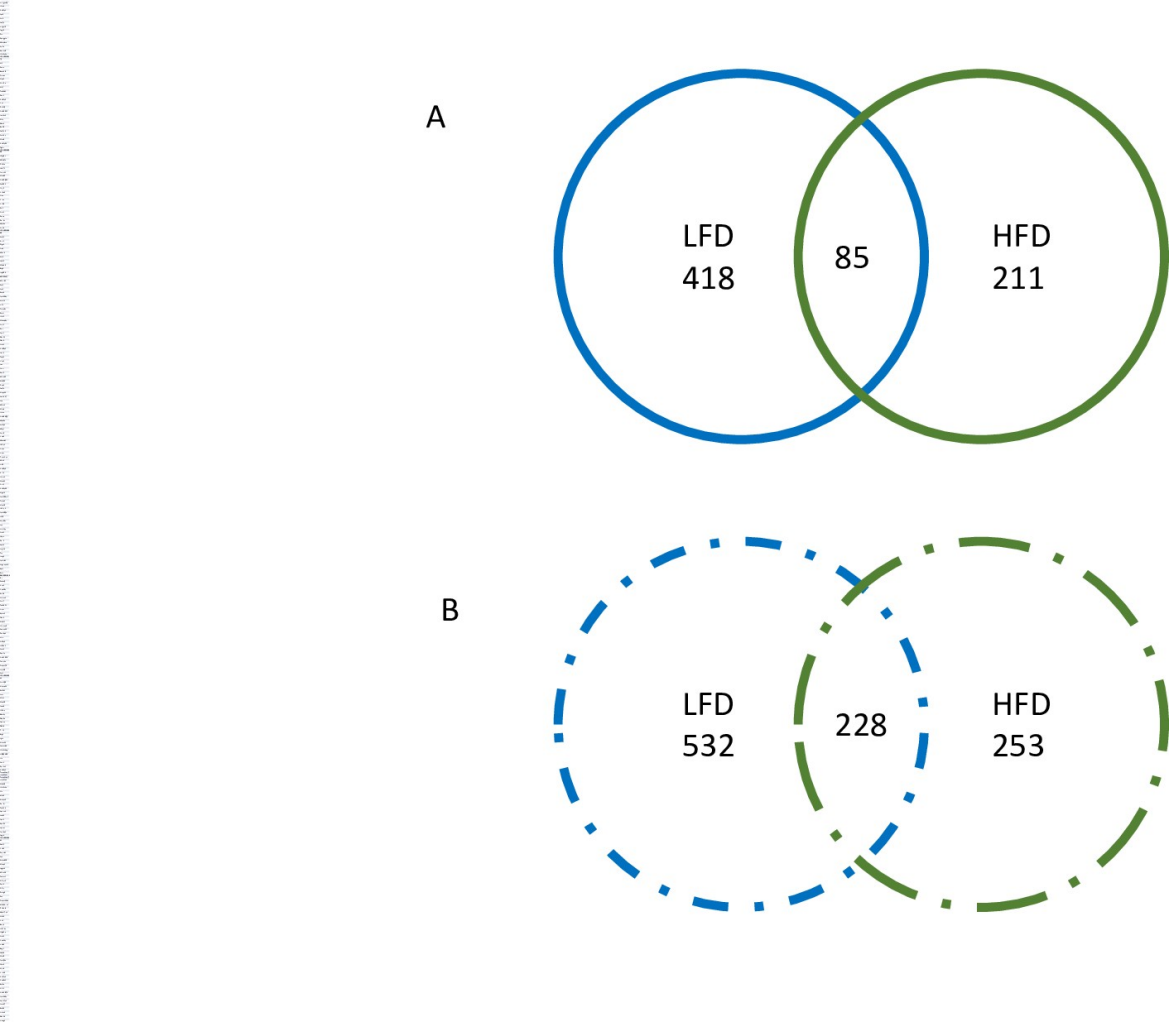
**Venn diagrams depicting A) shared and exclusively upregulated genes in TMG compared to IMG and B) shared and exclusively downregulated genes in TMG compared to IMG**.

**Fig 6:**
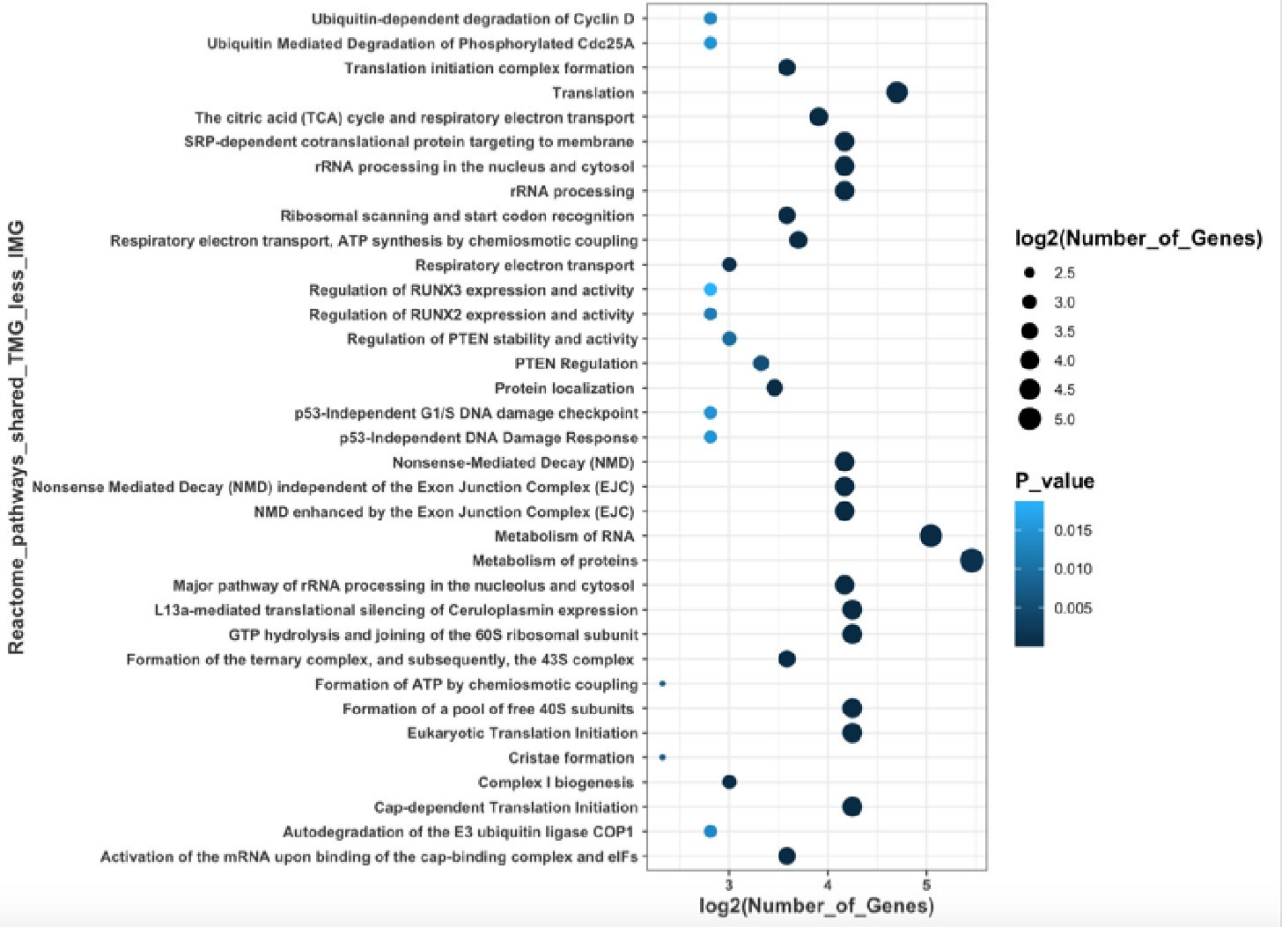
Reactome pathways that were downregulated in the TMG when compared to IMG in both LFD and HFD fed dams.

Using the bioinformatics and evolutionary genomics Venn diagram software (**Fig 5**), we took our two comparisons, TMG and IMG LFD dams, and TMG and IMG HFD dams, and compared genes that were up or downregulated to determine shared genes that were differentially expressed as well as diet specific pathways affected. There were 228 genes commonly downregulated in the TMG in comparison to the IMG irrespective of diet. Notable GO processes downregulated in the TMG of both LFD and HFD dams included amide biosynthetic process (GO:0043604; p <<< 0.0001) and translation (GO:0006412; p <<< 0.0001), as well as multiple processes involved in purine ribonucleotide and nucleoside biosynthesis (**S9 Table**) Notable molecular functions that were downregulated in the TMG compared to the IMG included structural constituent of ribosomes (GO:0003735; *p* <<< 0.0001), U1 snRNP binding (GO:1990446; *p* < 0.01), and NADH dehydrogenase activity (GO:0050136,0008137,0003954; p < 0.05). Reactome pathways that were significantly downregulated in the TMG compared to the IMG included translation (R-MMU-72766; p <<< 0.0001), formation of a pool of free 40S subunits (R-MMU-72689; p <<< 0.0001), and nonsense mediated decay (R-MMU-975957 and R-MMU-927802; p <<< 0.0001), as well as multiple pathways related to rRNA processing, translation, and initiation (**S10 Table**). There were 85 genes exclusively upregulated in the TMG compared to IMG in dams irrespective of diet; however, no notable GO biological processes or reactome pathways were common amongst them. **Fig 6** depicts reactome pathways that were downregulated in the TMG compared to IMG in both LFD and HFD fed dams. Interestingly, in this study we observed that small RNAs such as *Snora73b*, *Rnu3b4*, and *Snord118* were differentially expressed between the TMG and IMG in both LFD and HFD fed dams, and is a topic addressed in our discussion.

Next we examined pathways that were exclusively up or downregulated by a specific dietary exposure. In LFD dams, there were 418 genes exclusively upregulated and 532 genes exclusively downregulated in the TMG in relation to the IMG. Molecular functions downregulated in TMG compared to IMG included those involved in DNA binding (GO:0043565; p < 0.001), DNA binding transcription activity (GO:0000981; p < 0.001), and double-stranded DNA binding (GO:0003700, p <0.001). Reactome pathways downregulated in the TMG compared to IMG related to membrane trafficking (R-MMU-199991; p < 0.01) and vesicle mediated transport (R-MMU-5653656; p < 0.01).

There were 211 genes exclusively upregulated and 253 genes exclusively downregulated in the mammary glands of HFD when comparing the TMG to IMG. Biological processes exclusively downregulated in the HFD TMG compared to IMG included ribosome biogenesis (GO:0042554, p < 0.05) and ribonucleoprotein complex biogenesis (GO:002613; p < 0.05).

## Discussion

This study supports the hypothesis that there are differences between the TMG and IMG in C57/BL6 mice during peak lactation. The stark changes observed within a diet between the TMG and IMG highlights the need for scientists to be vigilant in carefully documenting and reporting which mammary glands are being used for mammary gland studies. Our lab has previously documented the importance of dietary intake in regulating mammary gland gene expression [9], but have not yet investigated the importance of mammary gland position in transcriptome expression profile. In our study, TMGs and IMGs in this study did not come from the same dams. Although this may be perceived as a weakness of our study, the purpose of our experiment was to investigate the general differences between the TMG and IMG regardless of their individual differences. Additionally, inbred mouse lines have very little variability within each strain due to their isogenicity; therefore, we do not believe that this impacted our results significantly [10, 11]. Because we do not have milk collected separately from these glands, we cannot determine whether milk composition between the TMG and IMG are different in these mice. However, we recognize that this is an important limitation of this initial study and these are studies to be conducted at a different date.

The most significant downregulated reactome pathway in the TMG when compared to the IMG was the metabolism of RNA (**S8;** p <<< 0.0001). One explanation for the differences in RNA metabolism might be due to noncoding RNAs such as small nuclear RNAs (snRNA) and small nucleolar RNAs (SnoRNA). In this study we observed that small RNAs such as *Snora73b*, *Rnu3b4*, and *Snord118* were significantly different between the TMG and IMG in both LFD and HFD fed dams. This is particularly of interest, as pathways related to nonsense mediated decay (NMD) were demonstrated to be the most significantly different between the TMG and IMG in shared pathways between LFD and HFD fed dams (p << 0.0001; **S10**). NMD eliminates eukaryotic RNAs with premature stop codons, and it has been demonstrated that genes that host large quantities of snoRNAs in their introns produce NMD sensitive splice variants [12]. In addition to ncRNAs having a role in NMD, these genes have a wide range of functions including ribosome biogenesis and splicing [13–15]. It has also been demonstrated that snoRNAs participate in methylation and pseudo-uridylation thereby regulating the expression of their host genes [16]. While there is little research regarding the specific small RNAs and their function with relation to lactation, multiple studies have demonstrated the importance of many of these small RNAs and physiologic function. For example, mutations in *snord118*, which is responsible for encoding the snoRNA U8, results in cerebral microangiopathy leukoencephalopathy [13]. Therefore, it is possible that small RNAs could be partially responsible for the vast differences we observed between the IMG and TMG in both diet groups. However, our study was not mechanistic in nature, and further experiments are required to examine individual small RNAs and their effects on transcription and translation in the lactating mammary gland.

Other pathways significantly affected by positional variation included translation and translation initiation. The regulation of translation and translation initiation is extremely crucial during lactation and in eukaryotes, mRNA translation is primarily controlled through translation initiation [17]. This is supported by the fact that we observed rRNA processing pathways to also be significantly altered between mammary gland position [18]. Lactation in and of itself is characterized by an increase in rate of translation, with lactating cows having at least a four-fold increase in mRNA translation rate when compared to non-lactating cows [19]. It has also been demonstrated that administration of growth hormone in cows promoted protein translation via initiation and elongation [20]. Thus, pathways related to translation were significantly altered by mammary gland position and is an interesting area of research when determining different physiological functions of mammary gland position. However, because milk from these glands were not taken, future research should be focused on how this may affect milk synthesis in an individual gland.

Interestingly, within each diet we observed differences in a multitude of insulin-like growth factor binding proteins (IGFBP), such as *Igfbp5*, *Igfbp2*, and *Igfbp3* between the TMG and IMG. In dams fed a LFD, we observed significant differences in insulin-like growth factor binding protein (IGFBP) genes such as *Igfbp5*, *Igfbp2*, and *Igfbp3*. The IGF axis is crucial in mammary gland physiology and play a role in mammary epithelial cell differentiation [21]. It is interesting to note that *Igfbp5* is upregulated during involution [22] and it has been hypothesized that *Igfbp5* may act as a tumor suppressor in breast cancer [21], indicating that mammary gland position may have an influence on timing of involution. *Igfbp2* is thought to have an opposite effect of *Igfbp5* [21], which is congruent with our findings which showed an increase in *Igfbp2* in the IMG and a decrease of *Igfbp2* in the IMG when compared to the TMG. *Igfbp2* is thought to have a pro-tumorigenic effect [23], potentially via an ER-alpha dependent mechanism [24]. Thus, the significant differences we observed in *Igfpb* expression between the TMG and IMG of dams fed a LFD indicate that there could be significant changes in mammary epithelial cell differentiation in dams fed a LFD between the TMG and IMG and may explain positional differences in breast cancer susceptibility between mammary gland position as well as the influence of mammary gland position on mammary involution.

This research highlights the drastic gene expression differences mammary gland position can have. We have shown here that mammary gland position has a significant effect on gene expression under either a HFD or a LFD consumption. Thus, it is vital for scientists to begin documenting and reporting the mammary glands used in mammary gland related studies. Since the main goal of this study was to identify the transcriptome changes between mammary glands collected from different locations, we employed pairwise comparisons within each diet. More studies using a statistical model accounting for the impacts of diet, mammary gland location and the interaction of the two are required to comprehensively dissect the impacts of biological and environmental factors on mammary gland function. Further studies are also needed to assess the effect of time dependent gene expression pattern in mammary gland as well.

## Materials and methods

### Animal care and diets

The University of Wisconsin-Madison Research Animal Care and Use Committee (A005789) approved all animal protocols and experiments. All experiments were conducted according to proper protocol guidelines and regulations. The animal facility was maintained at 25°C at 50–60% humidity under a 12:12 light: dark cycle for the entirety of the study. Ten 3-week old WT C57BL/6 mice were randomized and divided into either a low-fat diet (LFD) or high fat diet (HFD) *ad libitum* (n = 3 LFD/TMG, n = 3 LFD/IMG, n = 3 HFD/TMG, n = 3 HFD/IMG) for a total of 12 unique mammary glands used for analysis (**S11 Table**). Mouse feed was changed on a weekly basis. The HFD (TD.06414; Envigo) consisted of 60% fat, 21.4% carbohydrates, and 18.3% protein at 5.1 kcal/g. The LFD (TD.2019; Envigo) consisted of 9% fat, 44.9% carbohydrates, and 19.0% protein at 3.3 kcal/g. Mice were on their respective diets for four weeks prior to mating. Female mice were bred at 7 weeks of age, with date of vaginal plug denoted as embryonic day zero (E0) and date of parturition as lactation day zero (L0). Litters were not standardized, but litters sizes were not significantly different between treatment groups (mean ± SE; LFD IMG = 6.7 ± 0.33, p = 0.10, HFD IMG = 8.3 ± 0.89, LFD TMG = 6.7 ± 0.33, HFD TMG 5.7 ± 0.67).

### Animal tissue collection and processing

Mice were euthanized the morning of lactation day 11 (L11; peak lactation) via CO_2_ asphyxiation and decapitation. Only one dam was euthanized at a time, to prevent mix ups in dams as well as to decrease suffering. Euthanasia chamber was cleaned prior and after use. For mice in all diet groups, either the entire right thoracic mammary gland or the entire right inguinal mammary gland was collected and snap frozen in liquid nitrogen for RNA extraction and sequencing. Thoracic and inguinal glands did not come from the same dam and is an issue that we have addressed in our discussion.

### Whole transcriptome RNA-sequencing

At total of 12 unique mammary glands underwent RNA extraction. Mammary gland RNA was extracted using the miRNeasy mini kit (Qiagen, Germany). RNA quantity was determined using a Qubit 3.0 fluorimeter (Invitrogen, Carlsbad, CA, USA). RNA quality was confirmed using the RNA 6000 Nano kit on the Agilent 2100 Bioanalyzer (Agilent Technologies, Santa Clara, CA, USA). All samples used for downstream analysis were of an RNA integrity score (RIN) of 7.8 or above. One microgram of total RNA per sample was used for RNA-sequencing library preparation. All samples were prepared using the TruSeq Stranded Total RNA sample preparation kit (Illumina, San Diego, CA, US) per manufacturer’s instructions. Quality and quantity of RNA libraries were subsequently evaluated using the DNA 1000 kit using the Agilent 2100 Bioanalyzer (Agilent Technologies, Santa Clara, CA, USA). Prepared cDNA libraries were normalized using the Kapa library quantification kit (Kapa Biosystems, Wilmington, MA, US). cDNA libraries were first sequenced using a MiSeq Nano kit (Illumina, San Diego, CA, US). Further normalization of pooled library was done according to the index ratio obtained by the MiSeq to ensure even sequencing depth among pooled samples. Finally, pooled samples were sequenced on the NextSeq500 (Illumina, San Diego, CA, US) using 150 cycle high-output kit to generate paired-end reads (2x75bp). Raw read quality was verified using FastQC. All data is available at https://zenodo.org/record/4025240#.X1vhnWdKhsM.

### Gene expression analysis

**Fig 7:**
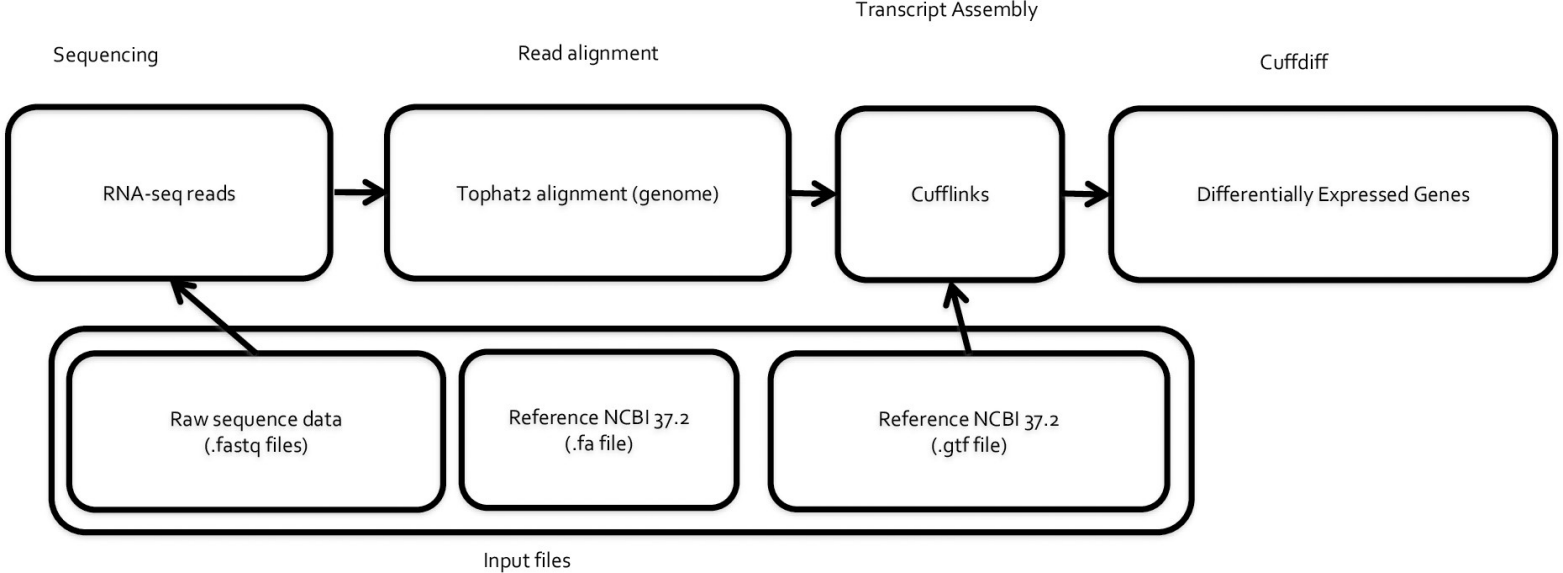
Data analysis workflow for transcriptomic analysis of C57BL/6 mammary glands at lactation day 11.

To align raw reads to the mouse genome, NCBI build 37.2 Mouse genome (https://ccb.jhu.edu/software/tophat/igenomes.shtml) was used as a reference and Tophat2 [25] was used to perform sequence alignment. Normalized read count for each gene, fragments per kilobase of transcript per million mapped reads (FPKM) was obtained using Cufflinks [26]. Genes with FPKM values <1 were excluded from further analysis. The main goal of this study is to identify the transcriptome changes between mammary glands collected from different locations. Pairwise, differential gene expression (DEG) analysis was performed using cuffdiff [27] for these two comparisons: 1) the DEGs between IMG and TMG when fed a LFD and 2) the DEGs between IMG and TMG when fed a HFD. Genes with adjusted-pvalue < 0.1 calculated by cuffdiff using Benjamini-Hochberg correction were considered significantly differentially expressed. Gene function annotation and reactome pathway analysis were performed using PANTHER [28, 29] using a custom background gene list, which was prepared by keeping the genes expressed (FPKM >0) in at least 10% of all the samples analyzed in this study. This filtering strategy allowed the identification of both a tissue specific background gene list and the retainment of potentially lowly expressed genes which could be of significant biological impact. Using the venn diagram tool created by the bioinformatics and evolutionary genomics website (http://bioinformatics.psb.ugent.be/webtools/Venn/), we compared common DEGs and exclusive DEGs between inguinal and thoracic mammary glands when mice were fed either a LFD or HFD. Workflow for the RNA-sequencing portion of the study is depicted in **Fig 7**. To identify stably expressed genes regardless of mammary gland location or diet, we performed ANOVA (using the scipy.stats package in Python) analysis using the FPKM values. P-value >0.5 and Coefficient of variation <0.2 were used as a cutoff to determine stably expressed genes [30]. Genes that were determined to be stably expressed in all samples are listed in **S12 Table**. We picked two of the stably expressed genes as reference genes in the RT-qPCR expression analysis.

### Quantitative PCR Validation

Primers were purchased from Integrated DNA Technologies (IDT, Coralville, IA). Primers were designed and verified using Primer-BLAST (https://www.ncbi.nlm.nih.gov/tools/primer-blast/). All primers followed MIQE guidelines [31] and all were verified via a standard curve and melt curve. All primers had an annealing temperature of 60°C, amplification efficiency between 95%-110%, no primer dimers, and had an R^2^ of 0.96 or above. All samples were normalized to 1μg and reverse transcribed to cDNA using the Applied Biosciences High-Capacity cDNA Reverse Transcription Kit (Foster City, CA, US). cDNA was diluted in RNase free H_2_O in a 1:5 ratio. Reaction mixtures contained 6.5μL of SSoFast EvaGreen Supermix (Bio-Rad, CA, US), 0.5μL of 10μM of each forward and reverse primers, 2.5μL of diluted cDNA, and 0.75μL of RNase free H_2_O. The following conditions were used: Step 1) 1 cycle at 95°C for 3 minutes, Step 2) 95°C for 10 seconds and Step 3) 60°C for 30 seconds. Steps 2 and 3 were repeated for 45 cycles. Quantitative real-time polymerase chain reaction (qRT-PCR) was conducted using a Bio-Rad CFX96 Touch Real-Time PCR Detection System (CA, US). Samples were run in duplicate, with a standard deviation less than 0.5. The geometric mean of Ribosomal Protein 9 (*RPS9*), Keratin 8 (*KRT8*; a luminal epithelial cell marker used to control for epithelial cell content) [32, 33], Eukaryotic elongation factor 2 (*Eef2)* and NADH:ubiquinone oxidoreductase subunit B10 (*Ndufb10*) was taken to represent the reference gene [34]. Fold change was calculated using the 2^-ΔΔCt^ method [35]. RPS9 and K8 have been used as the reference gene in the experiments run in our laboratory using mouse mammary glands [32]. Additionally, Eef2 and Nduf10 were determined to be stably expressed in our samples and therefore we deemed using the four of them appropriate to use as our reference genes. However, we did not validate the reference genes using Normfinder or GeNorm and is a limitation of this study. Sequences of genes used to verify qRT-PCR results are located in **S13 Table**.

### Statistical analysis

qRT-PCR was evaluated using a student’s t-test in GraphPad Prism v7 (San Diego, CA, US). Heat maps were generated in GraphPad Prism using the most significant DEGs. Reactome pathway figures were generated in R (Berkeley, CA).

## Supporting information

S1 Table Differentially expressed genes between TMG and IMG of LFD fed dams. 1264 DEGs when comparing thoracic and inguinal mammary glands of LFD fed L11 dams.
(XLSX)

S2 Table GO biological processes significantly upregulated in the thoracic mammary glands of LFD fed dams. (XLSX)

S3 Table GO biological processes significantly downregulated in the thoracic mammary gland of L11 dams fed a LFD. (XLSX)

S4 Table GO molecular functions significantly altered between TMG and IMG of LFD fed dams. GO Molecular Functions upregulated in thoracic mammary glands in L11 dams fed a LFD (top). GO Molecular Functions downregulated in thoracic mammary glands in L11 dams fed a LFD (bottom).
(XLSX)

S5 Table Reactome pathways significantly downregulated in the thoracic mammary glands of L11 dams fed a LFD. (XLSX)

S6 Table DEGs between TMG and IMG of HFD fed dams. 777 DEGs between the thoracic and inguinal mammary glands L11 dams fed a high fat diet.
(XLSX)

S7 Table GO biological processes significantly downregulated in the thoracic mammary glands of L11 dams fed a HFD. (XLSX)

S8 Table Reactome pathways significantly downregulated in the thoracic mammary glands of L11 dams fed a HFD. (XLSX)

S9 Table GO biological processes significantly downregulated in the thoracic mammary gland regardless of dietary exposure. (XLSX)

S10 Table Reactome pathways significantly downregulated in the thoracic mammary glands regardless of dietary exposure. (XLSX)

S11 Table Animal numbers and mammary gland used for analysis. (XLSX)

S12 Table Genes that were determined to be stably expressed in all samples. (XLSX)

S13 Table Primer pairs used in qRT-PCR verification. (XLSX)
